# Torsion of the gallbladder: a case report

**DOI:** 10.1186/1752-1947-2-237

**Published:** 2008-07-24

**Authors:** Samia Ijaz, Kaji Sritharan, Neil Russell, Manzoor Dar, Tahir Bhatti, Michael Ormiston

**Affiliations:** 1 Hemel Hempstead NHS Trust, Hillfield Road, Hemel Hempstead, HP2 4AD, UK

## Abstract

**Introduction:**

Torsion of the gallbladder is a rare condition that most commonly affects the elderly. Pre-operative diagnosis is the exception rather than the rule. Any delay in treatment can be fatal as the gallbladder may rupture, leading to biliary peritonitis.

**Case presentation:**

We present the case of an 80-year-old woman who was admitted with right upper quadrant pain initially thought to be secondary to acute cholecystitis. Subsequent ultrasound and computed tomography scans of the abdomen revealed signs suggestive of acute cholecystitis but neither modality detected any gallstones. As the patient's symptoms failed to resolve on conservative management, she was taken to theatre for an open cholecystectomy. Intra-operatively, the gallbladder had undergone complete torsion and appeared gangrenous. A routine cholecystectomy followed and she recovered from the operation without incident.

**Conclusion:**

It is rare to diagnose torsion of the gallbladder pre-operatively despite advances in diagnostic imaging. However, this differential diagnosis should be borne in mind particularly in the elderly patient, without proven gallstones, who fails to improve on conservative management. An emergency cholecystectomy is indicated in the event of diagnosing torsion of the gallbladder to avert the potentially lethal sequelae of biliary peritonitis.

## Introduction

Torsion of the gallbladder is an extremely rare clinical entity that was first described by Wendel in 1898 [1]. The incidence of this condition appears to be on the increase and this is possibly related to an increasingly aging population.

We present the case of an 80-year-old woman who was admitted with symptoms and signs of presumed cholecystitis. Her symptoms did not resolve on conservative management and she was taken to theatre for an open cholecystectomy. Intra-operatively, the authors observed that the gallbladder had undergone torsion leading to gangrene.

## Case presentation

An 80-year-old woman presented to the emergency department with a 24-hour history of sudden onset, severe right upper quadrant pain. The pain was sharp and constant in nature. It was relieved by sitting up and exacerbated by movement and deep inspiration. She felt nauseous but had not vomited and her bowels had opened normally the day before. Her past surgical history included an appendicectomy, a hysterectomy and bilateral salpingo-oophorectomy and a left inguinal hernia repair. In addition, she suffered from hypertension and osteoarthritis.

**Figure 1:**
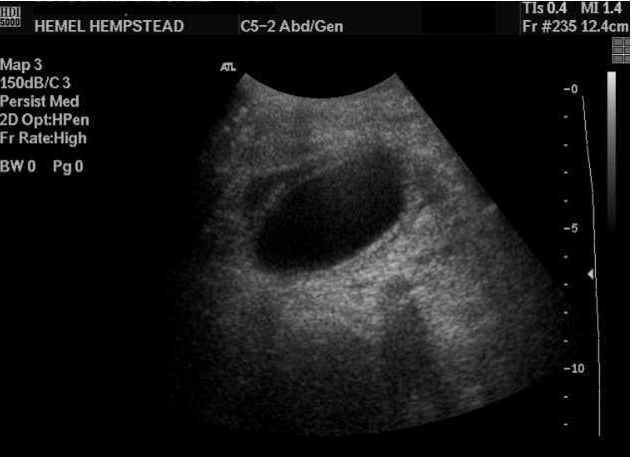
**Abdominal ultrasound**. A distended, thick-walled gallbladder with no gallstones and a cuff of pericholecystic fluid were revealed.

**Figure 2:**
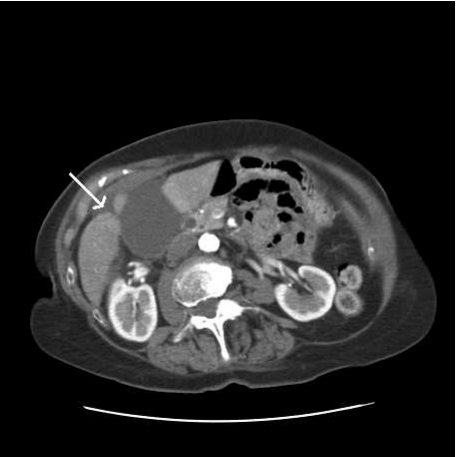
**Abdominal computed tomography scan**. Focal thickening of gallbladder neck, a hugely distended and inflamed gallbladder as well as fluid in the anterior hepatic space (as indicated by the arrow) can be seen.

On examination, she was afebrile and her vital signs were all within normal limits. Abdominal examination revealed a tender mass in the right upper quadrant (Figures 1 and 2). Her white cell count was raised at 12.85 × 10^9^/litre, with a neutrophil count of 10.3 × 10^9^/litre. The rest of her blood test results were entirely normal, including liver function tests. An ultrasound scan of her abdomen was organised and this showed a distended gallbladder with a thickened wall suggestive of cholecystitis. However, no stones were seen and there was no intra or extrahepatic biliary duct dilatation.

Her clinical picture did not improve despite intravenous antibiotics and fluids so an abdominal computerised tomography (CT) scan was carried out. CT demonstrated focal thickening around the neck of the gallbladder as well as a small amount of pericholecystic fluid that had extended into the right anterior perihepatic space.

As the patient's condition was not improving (her white cell count had also increased to 15.4 × 10^9^/litre) she was scheduled for an open cholecystectomy, as the surgeon was more familiar with the open rather than the laparoscopic approach. At operation, there was free, bile-stained fluid on opening the peritoneal cavity and the gallbladder was gangrenous and grossly distended. On closer scrutiny, the gallbladder had undergone a complete anticlockwise torsion. A routine cholecystectomy followed the initial detorsion and decompression. The patient recovered without incident and was discharged from hospital within a week.

## Discussion

Torsion of the gallbladder occurs when the gallbladder rotates on its mesentery along the axis of the cystic duct and cystic artery, consequently compromising its blood supply and obstructing biliary drainage. It is most common in elderly women, usually in the seventh and eighth decades of life. A pre-operative diagnosis is unusual and prompt surgery is necessary to avoid the high morbidity and mortality associated with gangrene and perforation [2].

Torsion can be complete (that is, more than 180°) or incomplete (less than 180°). Anatomical anomalies can result in a gallbladder that is suspended on an abnormally long mesentery, allowing it to hang freely from the liver bed and consequently making it more susceptible to rotational instability. Torsion is thought to occur more frequently in the elderly due to the loss of visceral fat and elasticity with advancing age, thus permitting the gallbladder to hang freely [2,3].

Given these anatomical aberrations, precipitating factors are also necessary to initiate torsion. Suggested factors include intense peristalsis of stomach, duodenum or transverse colon, spinal deformities and tortuous atherosclerotic cystic arteries (acting as rigid fulcrums for torsion). Gallstones are unlikely to cause torsion, as they are only present in 20% to 33% of affected patients. Most patients develop a clockwise rotation [4]. There are suggestions in the literature that gastric peristalsis promotes clockwise torsion and colonic peristalsis facilitates counter clockwise torsion, but evidence is somewhat lacking.

In incomplete torsion the patient frequently presents with symptoms similar to recurrent biliary colic, but patients with complete torsion generally present with a short history of sudden onset, severe right upper quadrant pain and vomiting. An abdominal mass may or may not be palpable and there are usually no signs of toxaemia or jaundice. Laboratory investigations reveal a normal or high white cell count and normal liver function tests as the common bile duct is not usually obstructed.

Ultrasonography and CT are the main imaging modalities that are employed in this context but it is rare for clinicians to make the diagnosis based on radiographic findings. However, ultrasound and CT can reveal a 'floating' gallbladder, without gallstones, lying transversely outside its anatomical fossa. The gallbladder neck may appear conical, corresponding to the twisted pedicle. Non-specific findings of gross wall thickening and distension are common to both torsion and calculous cholecystitis [5]. Magnetic resonance cholangiopancreatography (MRCP) may also aid the diagnosis pre-operatively. MRCP can show a V-shaped distortion of the extrahepatic bile ducts due to traction by the cystic duct, a tapering and twisting interruption of the cystic duct, a distended gallbladder and a high signal intensity within the gallbladder wall on T1-weighted images, suggesting haemorrhage and necrosis [6].

Prompt laparoscopy or laparotomy followed by detorsion and cholecystectomy is mandatory to avert the potentially fatal sequelae of gangrene and perforation. Laparoscopic cholecystectomy is both feasible and safe, in experienced hands. Initial decompression of the distended gallbladder allows for easier handling in both open and laparoscopic approaches.

## Conclusion

In summary, torsion of the gallbladder is rare and very difficult to diagnose pre-operatively despite advances in diagnostic imaging. Nonetheless, this diagnosis should be considered in all elderly patients presenting with symptoms suggestive of acute cholecystitis, particularly in the absence of gallstones.

## Abbreviations

CT: computed tomography; MRCP: magnetic resonance cholangiopancreatography.

## Competing interests

The authors declare that they have no competing interests.

## Authors' contributions

All of the named authors were involved in the preparation of this manuscript. All authors read and approved the final manuscript.

## Consent

Written informed consent was obtained from the patient for publication of this case report and any accompanying images. A copy of the written consent is available for review by the Editor-in-Chief of this journal.
